# Cytokine Imbalance as a Common Mechanism in Both Psoriasis and Rheumatoid Arthritis

**DOI:** 10.1155/2017/2405291

**Published:** 2017-01-25

**Authors:** Yong Tan, Qiu Qi, Cheng Lu, Xuyan Niu, Yanping Bai, Chunyan Jiang, Yang Wang, Youwen Zhou, Aiping Lu, Cheng Xiao

**Affiliations:** ^1^Institute of Basic Research in Clinical Medicine, China Academy of Chinese Medical Sciences, Beijing 100700, China; ^2^Institute of Clinical Pharmacology, Beijing An Zhen Hospital, Capital Medical University, Beijing 100029, China; ^3^Department of Dermatology, China-Japan Friendship Hospital, Beijing 100029, China; ^4^Department of Dermatology, Beijing Hospital of Traditional Chinese Medicine, Beijing 100010, China; ^5^Department of Dermatology and Venerology, Peking University First Hospital, Beijing 100034, China; ^6^Department of Dermatology and Skin Science, University of British Columbia, Vancouver, BC, Canada; ^7^Molecular Medicine Laboratory and Chieng Genomics Center, Vancouver Coastal Health Research Institute, Vancouver, BC, Canada; ^8^Institute for Advancing Translational Medicine in Bone & Joint Diseases, School of Chinese Medicine, Hong Kong Baptist University, Kowloon Tong, Hong Kong; ^9^E-Institute of Chinese Traditional Internal Medicine, Shanghai Municipal Education Commission, Shanghai 201203, China; ^10^Institute of Clinical Medicine, China-Japan Friendship Hospital, Beijing 100029, China

## Abstract

Psoriasis (PS) and rheumatoid arthritis (RA) are immune-mediated inflammatory diseases. Previous studies showed that these two diseases had a common pathogenesis, but the precise molecular mechanism remains unclear. In this study, RNA sequencing of peripheral blood mononuclear cells was employed to explore both the differentially expressed genes (DEGs) of 10 PS and 10 RA patients compared with those of 10 healthy volunteers and the shared DEGs between these two diseases. Bioinformatics network analysis was used to reveal the connections among the shared DEGs and the corresponding molecular mechanism. In total, 120 and 212 DEGs were identified in PS and RA, respectively, and 31 shared DEGs were identified. Bioinformatics analysis indicated that the cytokine imbalance relevant to key molecules (such as extracellular signal-regulated kinase 1/2 (ERK1/2), p38 mitogen-activated protein kinase (MAPK), tumor necrosis factor (TNF), colony-stimulating factor 3 (CSF3), interleukin- (IL-) 6, and interferon gene (IFNG)) and canonical signaling pathways (such as the complement system, antigen presentation, macropinocytosis signaling, nuclear factor-kappa B (NF-*κ*B) signaling, and IL-17 signaling) was responsible for the common comprehensive mechanism of PS and RA. Our findings provide a better understanding of the pathogenesis of PS and RA, suggesting potential strategies for treating and preventing both diseases. This study may also provide a new paradigm for illuminating the common pathogenesis of different diseases.

## 1. Introduction

Psoriasis (PS) and rheumatoid arthritis (RA) are immune-mediated chronic inflammatory diseases. PS is characterized by epidermal hyperplasia, and the predominant pathological feature of RA is the destruction of synovial joints. Studies have increasingly suggested that patients suffering from PS or RA have similarly increased risks of certain disorders, such as major adverse cardiovascular events, malignancy, and liver fatty changes, compared to the general population [1–6]. Additionally, these two diseases have a similar pathogenesis. Previous studies have indicated that the chronic inflammation mediated by T helper (Th) 17 and Th1 cells plays a key role in PS [7, 8]. Cytokines, including Th1-related (tumor necrosis factor- (TNF-) *α*, interferon gamma (IFN*γ*), and interleukin- (IL-) 2) and Th17-related (IL-17A, IL-17F, IL-22, IL-26, and TNF-*α*) proteins, together with IL-23, IL-20, and IL-15 were increased in the sera of PS patients [8, 9]. For RA, the concerted interaction of proinflammatory cytokines also plays a dominate role in its pathogenesis [10]. An increasing number of clinical and histopathological features of PS and RA are explained by an imbalance in particular cytokines, which is one of the most fascinating research topics inspiring researchers [9]. The similarities between the mechanisms involved in PS and RA imply an underlying genetic homogeneity of these two diseases. By exploring the genetic overlap of PS and RA, we seek to provide a better understanding of their molecular correlation and their shared mechanisms. The genetic commonality of PS and RA may provide increasing evidence for developing combined treatment targets for both diseases. These targets will eventually complement the traditional systemic treatments and biological agents that are currently available.

Due to the progress in high-throughput techniques for biological research, next-generation sequencing (NGS) platforms are often used to explore the gene profile; these platforms have the advantages of greater sensitivity and more precise quantification, thus providing a more complete picture of the transcriptome in studies of gene expression than that obtained by microarrays [11]. Measurements of mRNA expression by RNA sequencing are valuable for identifying the molecular changes that occur in cells, thus providing clues regarding the molecular networks involved in disease processes [12]. Studies have focused on molecular changes in PS or RA independently using transcriptome or gene expression profile technology [12, 13], but few reports have been published concerning the correlations between PS and RA at the transcriptome level, including in-depth studies of the mechanisms and molecular networks involved in the pathogenesis common to RA and PS.

The present study applied RNA sequencing technology to the peripheral blood mononuclear cell (PBMC) RNA of PS and RA patients and healthy volunteers, and differentially expressed genes (DEGs) were explored among the groups. Furthermore, bioinformatics analysis was performed to identify the key molecules and signaling pathways relevant to RA and PS as well as the upstream regulators related to the identified genes. This study aimed to obtain a comprehensive understanding of the cytokine imbalance in RA and PS based on DEGs, which may provide new insights into the pathogenesis of and suitable prevention strategies for these two diseases.

## 2. Materials and Methods

### 2.1. Patients

PS patients, RA patients, and healthy volunteers were recruited from the China-Japan Friendship Hospital in Beijing City of China at the dermatology clinic, the rheumatology clinic, and the health screening center, respectively. The diagnosis of PS was consistent with the guidelines of the care for the management of psoriasis from the American Academy of Dermatology and the guidelines for the treatment of psoriasis from the Psoriasis Study Group of Chinese Medical Association [14, 15]. Additionally, enrolled patients had no symptoms or signs of psoriatic arthritis. These patients had a psoriasis area severe index (PASI) greater than 10 or body surface area (BSA) greater than 10% but a PASI less than 30 and BSA less than 30%. A diagnosis of RA was based on the 1987 American College of Rheumatology revised criteria and the 2010 American College of Rheumatology/European League against Rheumatism classification criteria for RA. Disease activity was assessed by the Disease Activity Score in 28 joints (DAS28). For inclusion, the control subjects could not have a history of an arthritic disorder and were subject to the same exclusion criteria as the PS and RA patients. Given that RA is two- to threefold more common in females than males, only females were chosen as the observed subjects in this study [16].

The following subjects were excluded: individuals who were ≥65 years old and ≤18 years old; individuals with complications, such as cardiovascular and cerebrovascular diseases, respiratory, digestive, urinary, and hematological diseases, metabolic syndrome, and mental disturbances; individuals who were pregnant, lactating, or who planned to become pregnant within a year; individuals who received topical treatments (such as corticosteroids or retinoic acid) within 2 weeks, systemic therapy within 4 weeks, or biological therapy within 12 weeks; PS patients with a concurrent RA diagnosis; and RA patients diagnosed with any type of PS.

In summary, 10 female PS patients, 10 female RA patients, and 10 female healthy controls were enrolled into this study. All protocols involving human subjects were approved by the ethics committee of the China-Japan Friendship Hospital (ethics ID: 2014-58), and informed consent was signed by all participants before the study began.

### 2.2. PBMC Isolation and Total RNA Extraction

In all, 3 mL peripheral fasting blood samples were collected from all subjects in the morning. PBMCs were isolated using density gradient centrifugation. Specifically, based on Ficoll-Hypaque gradient solution (Histopaque-1077, Sigma-Aldrich, USA), 3 mL of heparinized whole blood was diluted to 6 mL with phosphate-buffered saline (PBS, pH 7.4), layered on top of 3 mL of Histopaque and centrifuged for 30 min at 400 ×g. PBMCs were aspirated, washed twice, suspended in PBS, and counted with a hemocytometer. PBMCs were lysed in Trizol reagent (1 mL/1 × 10^7^ PBMCs) (Invitrogen, Karlsruhe, Germany; Carlsbad, CA) and stored at −80°C for the subsequent testing. Total RNA in PBMC sample was isolated using the Trizol extraction method, and it was quantified with a NanoDrop ND-1000 spectrophotometer (Thermo Fisher Scientific Inc., Marietta, OH, USA). The RNA Integrity Number was greater than 7.0, and acceptable quality values accorded with A260/A280 ratios ranging from 1.8 to 2.2 for each total RNA sample.

### 2.3. Identification of DEGs

Total RNA of each sample was purified by adsorption of biotin oligo magnetic beads. cDNA synthesis was conducted after the binding of mRNA. Double-stranded cDNA was introduced to the cDNA fragment digested by NlaIII endonuclease, and the bound fragments contained CATG sites and adjacent poly A tails at the 3′ end. After precipitation of the 3′ cDNA fragment, Illumina adaptor 1 was added to the 5′ end. Both the adaptor 1 and CATG sites are recognized by MmeI, which cuts at a downstream CATG site and produces fragments of 17-bp tags with adaptor 1. Adaptor 2 was added to the 3′ end of these tags after the fragment was removed using beads attached to the 3′ end. Then, these sequences were prepared for Solexa sequencing [17].

Clean tags were produced by filtering the adaptor sequences and removing low-quality sequences (containing ambiguous bases). Only the tags with perfect matches or one mismatch were further considered and annotated based on the reference genes. The expression level of each gene was estimated by the frequency of clean tags and then normalized to TPM (number of transcripts per million clean tags), which is a standard method extensively used in DEG analysis [18]. The number of tags mapped to a given gene represented the expression level of this gene. Expression levels of a gene from two different samples were compared to provide an expression difference. Significance values for differences in expression were determined using a modified exact test. The gene was classified as differentially expressed only when the expression difference was greater than 1.2-fold with a *p* value less than 0.01.

### 2.4. Bioinformatics Analysis about DEGs

The information of shared DEGs identified in PS and RA was uploaded into the Ingenuity Pathways Analysis system (IPA, Ingenuity Systems, http://www.ingenuity.com). The “Core Analysis” module in IPA was utilized to analyze and visualize interactions of the shared DEGs. These interactions were characterized by specific canonical pathways and molecular networks. Analytical score was the negative base 10 logarithm of Fisher's exact test *p* value in canonical pathway analysis. Significance for biological functions of each network was symbolized by a *p* value for the enrichment of the genes in the network by comparison with the entire Ingenuity Pathway Knowledge Base.

## 3. Results

### 3.1. Baseline Characteristics of Study Subjects

The characteristics of the enrolled subjects, including age, disease duration, BMI, PASI, BSA, and results of blood routine and biochemical tests, are presented in Table 1. No significant differences in any of the examination indicators were noted among the groups.

### 3.2. Identified Shared DEGs between PS and RA and the Corresponding Functions

**Figure 1:**
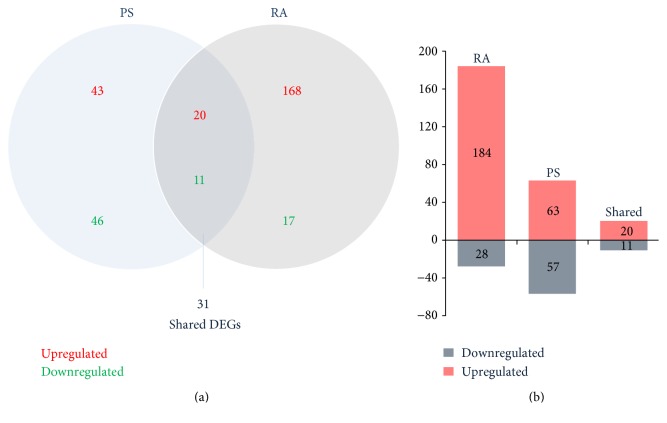
Number of DEGs in PS and RA. (a) The Venn diagram indicates the number of uniquely upregulated (red) or downregulated (green) genes from the comparisons of PS and RA with control and the number of shared DEGs. (b) The bar diagram shows the number of DEGs in PS, RA, and the shared DEGs between them.

**Figure 2:**
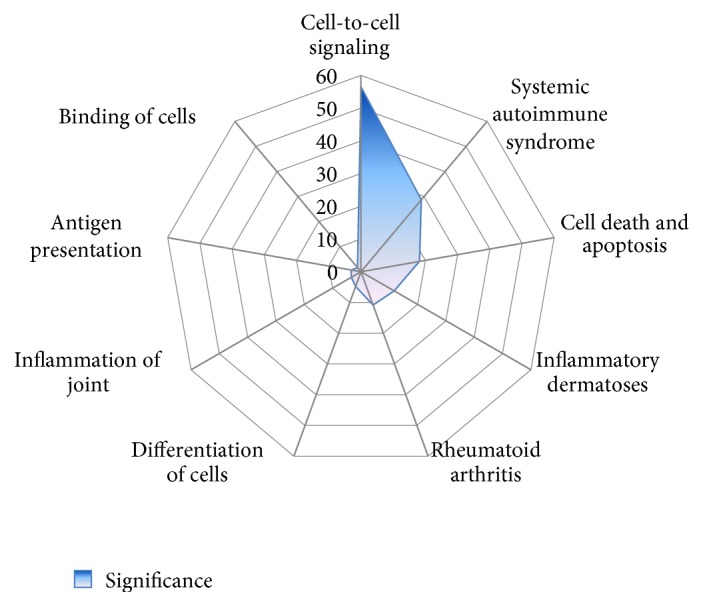
Biological functions corresponding to the shared DEGs.

One hundred and twenty genes in PS and 212 genes in RA were identified as DEGs when compared with the controls (Figure 1, Tables S1 and S2 in Supplementary Material available online at https://doi.org/10.1155/2017/2405291). As shown in Figure 1 and Table 2, there were 31 shared genes between PS and RA, including 20 upregulated and 11 downregulated DEGs, which reflects the complex association of PS and RA at the transcriptome level. The biological functions corresponding to the shared DEGs mainly include cell-to-cell signaling, systemic autoimmune syndrome, cell death and apoptosis, inflammatory dermatoses, and rheumatic arthritis (Figure 2, Table S3).

### 3.3. Networks of the Shared DEGs and the Corresponding Functions

**Figure 3:**
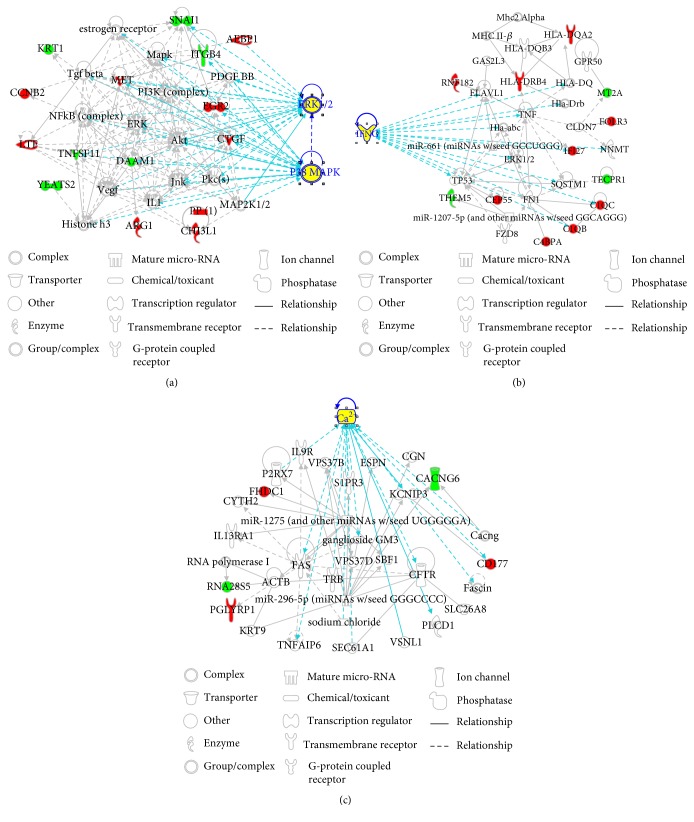
Biomolecular networks related to the shared DEGs. In each network, molecules are represented as nodes, and the biological relationship between two nodes is represented as a line. Red symbols represent upregulated DEGs; green symbols represent downregulated DEGs. Yellow symbols indicate the highly linked molecules from the Ingenuity Knowledge Database. Solid lines between molecules indicate a direct physical relationship between molecules, whereas dash lines represent indirect functional relationships. (a) The first network. (b) The second network. (c) The third network.

To reveal the connections between the shared DEGs, the biomolecular networks of these DEGs were constructed using IPA. As shown in Figure 3, these DEGs were associated with one another directly or indirectly, and three networks were established. Highly linked molecules of the networks included extracellular signal-regulated kinase 1/2 (ERK1/2), p38 mitogen-activated protein kinase (MAPK), interferon gene (IFNG), and Ca^2+^. The network functions included organismal injury and abnormalities, cell death and survival, and cellular function and maintenance.

### 3.4. Signaling Pathways Relevant to the Merged Bionetwork

**Figure 4:**
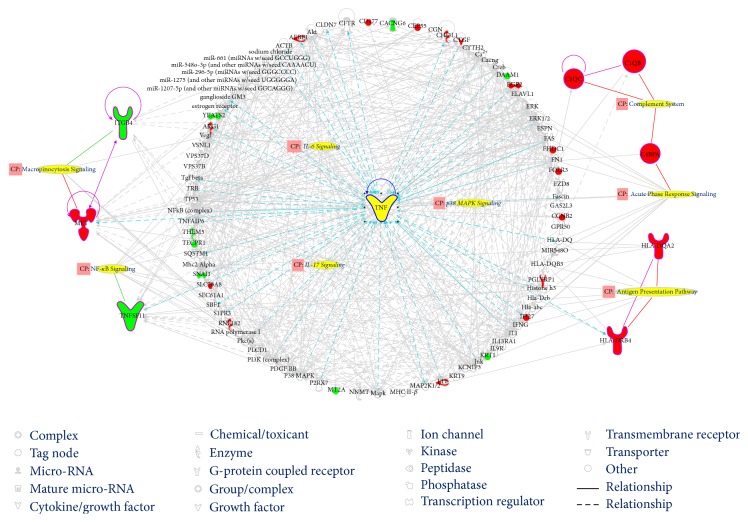
The most significantly relevant pathways and highly linked molecules relevant to the merged bionetwork. In the network, molecules are represented as nodes, and the biological relationship between two nodes is represented as a line. Red symbols represent upregulated DEGs; green symbols represent downregulated DEGs. Yellow symbols indicate the highly linked molecules and signaling pathways from the Ingenuity Knowledge Database. “CP” is an abbreviation of “canonical pathway,” which represents signaling pathways related to the merged bionetwork. Solid lines between molecules show a direct physical relationship between molecules, whereas dash lines show indirect functional relationships.

**Figure 5:**
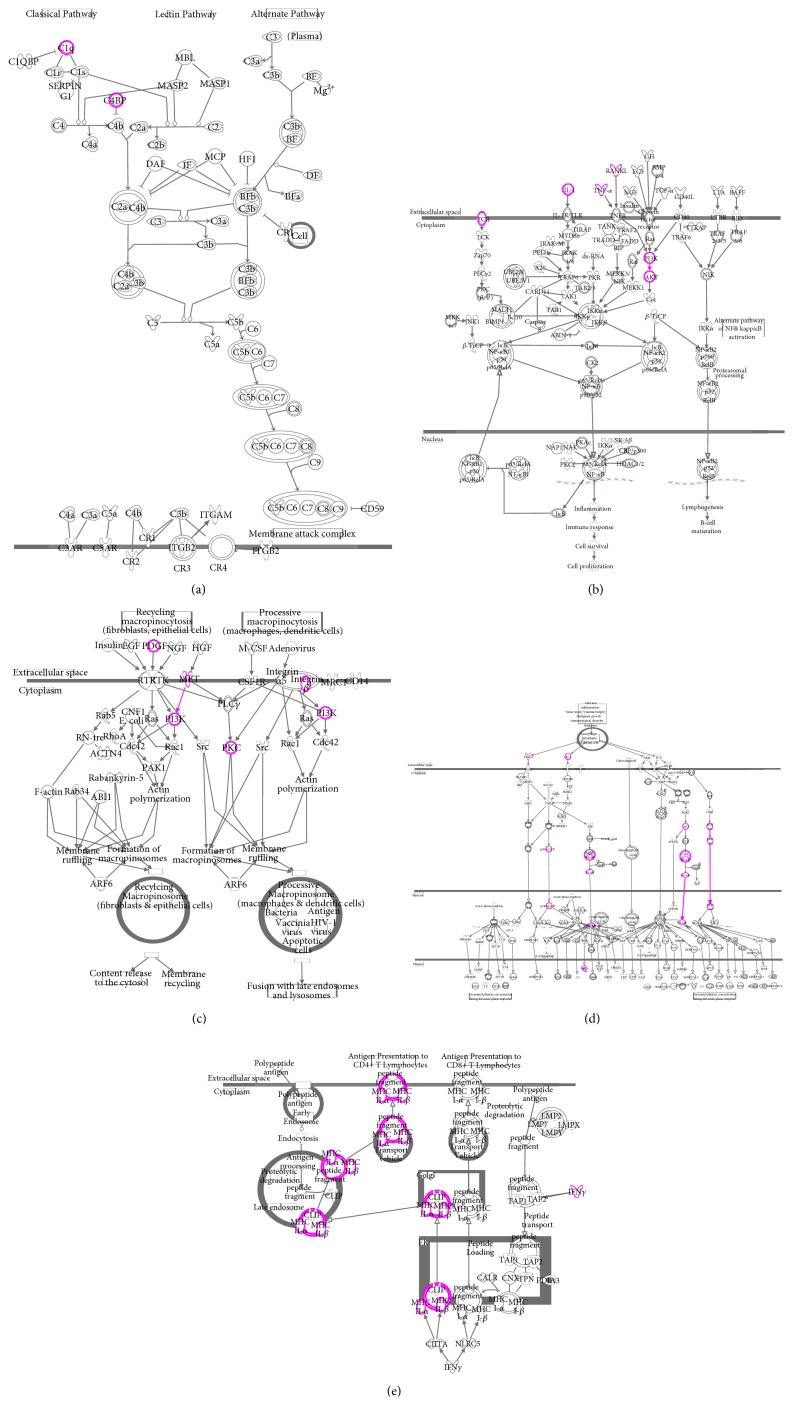
Top five pathways. Purple symbols represent the shared DEGs. (a) Complement system. (b) NF-*κ*B signaling. (c) Macropinocytosis signaling. (d) Acute phase response signaling. (e) Antigen presentation.

The three networks were merged and formed a large network that was associated with 42 signaling pathways. TNF was highly linked molecule of the network. The main categories corresponding to these signaling pathways were cytokine signaling, cellular immune response, and humoral immune pathways. Signaling pathways with −log(*p*-value) more than 2.00*E* + 00 represented the most significantly relevant pathways related to the merged network and included the complement system, antigen presentation, macropinocytosis signaling, acute phase response signaling, nuclear factor-kappa B (NF-*κ*B) signaling, IL-6 signaling, IL-17 signaling, and p38 MAPK signaling (Figures 4 and 5 and Table S4). Top five pathways of these pathways were associated with the shared DEGs.

### 3.5. Upstream Regulators of the Shared DEGs

**Figure 6:**
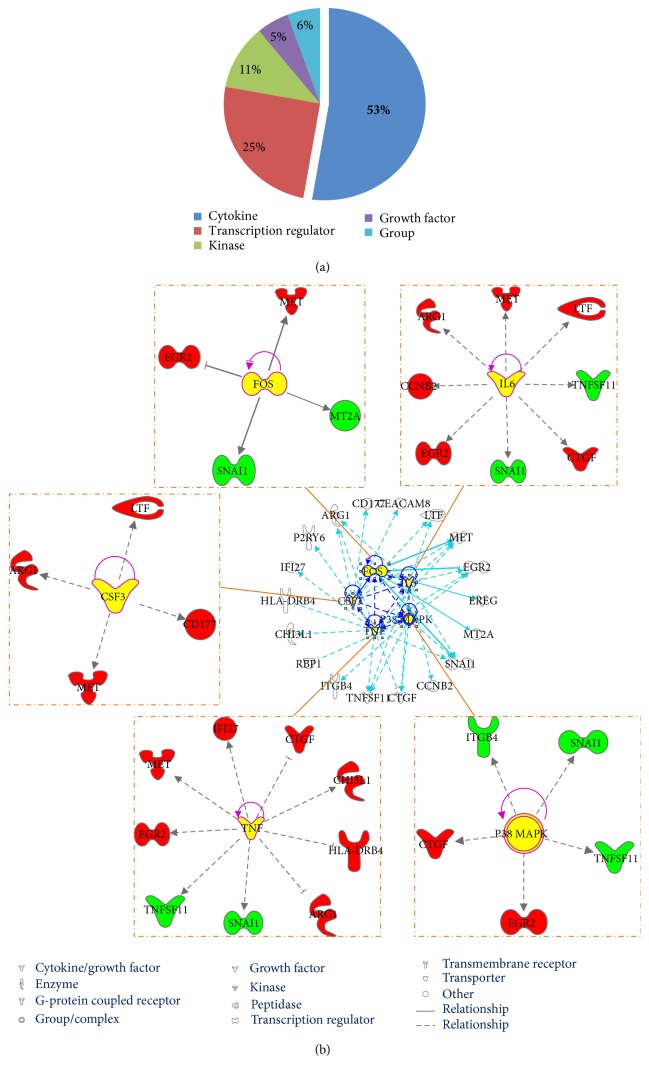
Upstream regulators relevant to the shared to DEGs. (a) The percentage of molecule type of upstream regulator. (b) Top five upstream regulators. Red nodes represent upregulated DEGs; green nodes represent downregulated DEGs. Yellow nodes represent the upstream regulators. Solid lines between molecules indicate a direct physical relationship between molecules, whereas dash lines indicate indirect functional relationships.

Thirty-six upstream regulators were identified by biomolecular network analysis, with the majority being cytokine molecules, that is, 52.78% of them (Figure 6(a), Table S5). The regulators with *p* values less than 1.00*E* − 04 included colony-stimulating factor 3 (CSF3), IL-6, FOS, p38 MAPK, and TNF, and the connection between every regulator and the corresponding target molecule is presented in Figure 6(b). The main biofunctions of the regulated effect networks corresponding to those regulators were inflammation or immune-related processes.

## 4. Discussion

**Figure 7:**
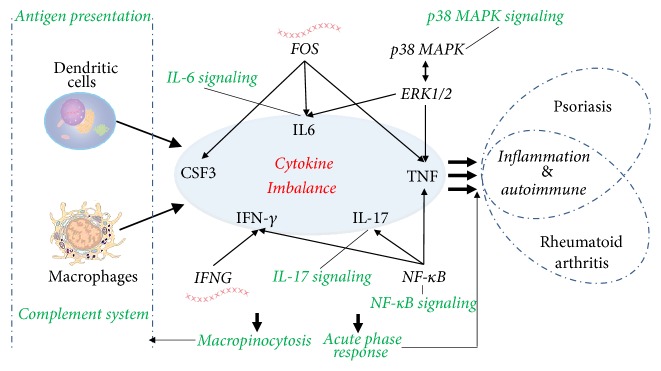
The common molecular mechanisms of PS and RA are characterized by a cytokine imbalance. Arrows represent the relationship between molecules or between molecules and cells. Green terms represent signaling pathways. The molecules in ovals are cytokines.

PS and RA are immune-mediated inflammatory diseases. An increasing number of studies have reported a correlation between PS and RA, but the exact common molecular mechanisms have not been elucidated. With the development of high-throughput detection and analysis techniques, including genomics and bioinformatics, the exploration of these comprehensive mechanisms has become feasible. PBMCs can be extracted from whole blood and consist of lymphocytes (T cells, B cells, and NK cells) and monocytes. Identifying gene expression in PBMCs is an important strategy to determine disease-specific genes in holism [19]. Methodologically, by comparison of gene profiles of patients with particular disease and healthy persons, the disease-specific genes can be found [20]. In this study, the PBMC gene profiles of PS patients, RA patients, and healthy volunteers were evaluated by determining the DEGs, and 31 gene expression signatures commonly shared between PS and RA were identified. Based on these shared DEGs, the pathogenesis common to both PS and RA was elucidated at the transcriptome level. The discoveries of this study suggest that the common mechanism of PS and RA mainly involves inflammation and an abnormal immune response characterized by a cytokine imbalance. Specifically, the identified highly linked molecules, significant signaling pathways, and upstream regulators were directly or indirectly associated with the regulation of a variety of cytokines. As shown in Figure 7, certain key molecules (ERK1/2, CSF3, FOS, IFNG, and TNF) and significant signaling pathways (the complement system, antigen presentation, macropinocytosis signaling, acute phase response signaling, NF-*κ*B signaling, IL-6 signaling, IL-17 signaling, and p38 MAPK signaling) were associated with an imbalance of cytokines; this imbalance may provide new clues for a better understanding of PS and RA.

The complement system is an essential component of innate immunity, and it plays an important role in modulating adaptive immunity. Its activation contributes to the pathogenesis of autoimmune and inflammatory diseases, such as PS and RA [21]. Reduction of complement activation is one of the mechanisms by which TNF-*α* inhibitors exert their effectiveness in these two diseases [22]. In this study, the upregulation of three DEGs (C4BPA, C1QB, and C4BPA) implied that the complement system was activated, which was consistent with previous studies. This study further confirmed that the complement system is indeed an attractive therapeutic target for both PS and RA. Professional antigen-presenting cells, such as dendritic cells (DCs), macrophages, and B cells, play a key role in triggering and/or maintaining the chronic inflammatory process in RA [23]. Increasing evidence indicates that RA treatment may occur through the manipulation of antigen presentation [24]. This study found that the activated antigen presentation pathway is characterized by upregulation of two DEGs, HLA-DQA2 and HLA-DRB4, and this upregulation is a common mechanism of PS and RA. Therefore, targeting antigen presentation may also be a new strategy for PS treatment. Macropinocytosis represents a distinct pathway of endocytosis in mammalian cells, and it significantly contributes to antigen presentation by the immune system. A study revealed that the type II collagen in an RA mice model was taken up by DCs and macrophages predominantly via inhibition of micropinocytosis [25]. In addition, the nonapoptotic cell death associated with perturbations of micropinocytosis is one apoptosis mechanism caused by RA [26]. This study showed that micropinocytosis signaling is also involved in PS, which offers a new method to understand the pathogenesis of PS. The acute phase response serves as a core of the innate immune response, and proteins relevant to this response were closely correlated with the development of RA [27]. The results from this study suggest that the pathogenesis of both PS and RA might partially result from the perturbation of the acute phase response. Regarding previous studies, the four signaling pathways discussed above are involved in the metabolism and regulation of cytokines. Specifically, when the complement system is stimulated by certain triggers, proteases in the system cleave specific proteins to release cytokines [28]. Antigen presentation plays an important role in cytokine production in PS and RA [29]. Cytokines, such as IFN*γ* and IL-17A, regulate macropinocytosis in macrophages [30]. Cytokine levels (IL-6 and IFN*γ*) mediate the acute phase response [31]. In short, perturbation of these pathways affected particular cytokines, which potentially partially reflects the pathogenesis of PS and RA.

IL-6 is a proinflammatory cytokine that induces activation of Th cells and controls the balance between Treg cells and Th17 cells. In lesional psoriatic skin, IL-6 is markedly elevated, and T lymphocytes encounter high IL-6 levels, thus allowing cutaneous T cells to avoid Treg suppression and increasing the Th17 inflammatory activity [32]. Targeting IL-6 signaling in PS may rebalance Treg/Th17 activity and ameliorate the disease [33]. IL-6 also stimulates the inflammatory and autoimmune processes in RA, and both deregulation of IL-6 production and blockade of IL-6 signaling are effective strategies in treating experimental models of RA [34]. IL-17 is the signature cytokine secreted by Th17 cells. IL-17 is particularly important in PS due to its proinflammatory effects and its involvement in an integrated inflammatory loop with DCs and keratinocytes, contributing to an overproduction of inflammatory cytokines that leads to amplification of the immune response [35, 36]. A study demonstrated that effective treatment of PS with TNF inhibitors was associated with suppression of IL-17 signaling [37]. Similarly, the therapeutic strategy of IL-17 signaling inhibition was also used to treat RA [38]. TNF, formerly known as TNF-*α*, is the best-known member of TNF superfamily. As a cytokine, TNF stimulates cell proliferation and cell differentiation and plays a key role in the pathogenesis of PS and RA [39]. A lack of the TNF G allele is associated with reduced PS severity [40]. CSF3 acts as a cytokine and may be produced by the endothelium and by macrophages. A previous study confirmed that CSF3 is a typical IL-17A-regulated gene in the keratinocytes of PS [35]. IFNG encodes the IFN*γ* protein. IFN*γ* is a cytokine secreted by Th cells (specifically, Th1 cells) and is an important activator of macrophages. IFN*γ* has been implicated in the initiation/maintenance of inflammation. A study showed that the pathogenesis of RA was correlated with reduced frequencies of IFN*γ* producers [41]. This study found that these particular cytokines, such as IL-6, IL-17, TNF, CSF3, and IFN*γ*, as well as the corresponding signaling pathways are involved in the pathogenesis of PS and RA, which not only verified previous discoveries but also reflected the importance of these cytokines. In brief, an imbalance in these cytokines plays a crucial role in the pathogenesis of PS and RA, and targeting these cytokines is a key strategy for prevention and treatment.

NF-*κ*B is essential for the expression of proinflammatory cytokines and controls a number of essential cellular functions, including the immune response, cell proliferation, and apoptosis. Loss of normal NF-*κ*B signaling regulation is a major contributor to a variety of inflammatory and autoimmune diseases, such as PS and RA [42, 43]. Activator protein-1 (AP-1) is recognized as a regulator of the expression of cytokines, such as CSF3, IL-6, and TNF, and is causally involved in PS and RA [44]. The immediate early gene product Fos is part of the AP-1 transcription factor, and its deregulation is associated with a variety of immunological defects. Selective inhibition of Fos function demonstrated that targeting Fos/AP-1 activity could be an promising new option for arthritis treatment [45]. ERK1/2 and p38 MAPK are important members of the MAPK family, which are responsive to inflammatory cytokines, and they are involved in cell differentiation, apoptosis, and autophagy. ERK1/2 is phosphorylated and activated via cell surface receptors stimulated by cytokines. Emerging data suggest that cytokine expression in response to p38 MAPK and ERK1/2 activation is involved in the etiopathogenesis of PS and that p38 MAPK signaling is an indicator of the loss of keratinocyte cell-cell adhesion in PS [46, 47]. IL-6, NF-*κ*B, and p38 MAPK signaling activation is an important characteristic of the inflammatory response in activated macrophages in RA, and p38 MAPK signaling is involved in the process of RA angiogenesis [48]. In addition, suppressing the expression of TNF-*α* and IL-6 through inhibiting the activation of NF-*κ*B and ERK1/2 is an important strategy for treating RA [49]. This study indicated that four biomolecules, NF-*κ*B, Fos, p38 MAPK, and ERK1/2, are highly linked molecules or upstream regulators that are closely associated with the shared DEGs in PS and RA and therefore regulated cytokines; these four biomolecules represent novel targets to prevent and treat these two diseases.

## 5. Conclusions

The common pathogenesis of PS and RA was characterized by a cytokine imbalance. The deregulation of certain key molecules, such as ERK1/2, CSF3, FOS, IFNG, and TNF, as well as the perturbation of signaling pathways, including the complement system, antigen presentation, macropinocytosis signaling, NF-*κ*B signaling, IL-6 signaling, IL-17 signaling, and p38 MAPK signaling, reflected this type of imbalance. The new findings in this study provided a new molecular understanding of PS and RA and could pave the road for the discovery of new strategies for treating PS and RA.

## Supplementary Material

In Supplementary Material, identified DEGs in PS vs. Control and RA vs. Control, functions and upstream regulators of the shared DEGs between PS and RA, as well as signaling pathways relevant to the merged bionetwork were listed in table in detail.

## Figures and Tables

**Table 1:** Characteristics of the enrolled subjects for the three groups.

Characteristic	Control (*n* = 10)	PS (*n* = 10)	RA (*n* = 10)
Age (years)	45.80 ± 3.50	48.60 ± 5.20	54.50 ± 7.10
Disease duration (years)	/	3.70 ± 1.20	2.10 ± 1.10
BMI (kg/m^2^)	22.34 ± 1.83	23.23 ± 4.71	25.81 ± 2.62
PASI	/	9.17 ± 8.56	/
BSA (%)	/	19.40 ± 4.14	/
ESR (mm/h)	/	/	39.14 ± 29.53
CRP (mg/L)	/	/	15.48 ± 15.40
RF (IU/mL)	/	/	63.76 ± 71.81
WBC (×10^9^/L)	4.92 ± 1.10	5.33 ± 0.98	6.09 ± 1.50
HGB (g/L)	126.20 ± 13.89	124.72 ± 11.54	122.38 ± 14.74
PLT (×10^9^/L)	228.70 ± 28.69	251.19 ± 34.56	246.75 ± 75.06

*Note*. Comparisons of clinical indicators of the PS group, RA group, and control group. An unpaired *t*-test was used for continuous variables analysis, and the data are expressed as the mean ± SD when appropriate (95% CI).

**Table 2:** The shared DEGs in PS and RA.

*n*	Symbol	Entrez gene name	Fold change	
			PS	RA
1	AEBP1	AE binding protein 1	2.615	2.644
2	ARG1	Arginase 1	1.295	2.224
3	C1QB	Complement component 1, q subcomponent, B chain	1.153	2.356
4	C1QC	Complement component 1, q subcomponent, C chain	1.177	3.000
5	C4BPA	Complement component 4 binding protein alpha	1.504	3.322
6	CACNG6	Calcium voltage-gated channel auxiliary subunit gamma 6	−1.329	−1.445
7	CCNB2	Cyclin B2	1.479	2.390
8	CD177	CD177 molecule	1.000	2.404
9	CEP55	Centrosomal protein 55	1.303	1.554
10	CHI3L1	Chitinase 3 like 1	1.095	1.258
11	CTGF	Connective tissue growth factor	4.700	4.954
12	DAAM1	Dishevelled associated activator of morphogenesis 1	−1.158	−1.489
13	EGR2	Early growth response 2	1.413	2.495
14	FHDC1	FH2 domain containing 1	1.122	1.631
15	FOLR3	Folate receptor 3 (gamma)	1.787	1.853
16	HLA-DQA2	Major histocompatibility complex, class II, DQ alpha 2	1.548	1.305
17	HLA-DRB4	Major histocompatibility complex, class II, DR beta 4	1.884	1.711
18	IFI27	Interferon alpha inducible protein 27	1.948	2.764
19	ITGB4	Integrin subunit beta 4	−1.531	−2.478
20	KRT1	Keratin 1	−1.142	−1.415
21	LTF	Lactotransferrin	1.526	1.708
22	MET	MET protooncogene, receptor tyrosine kinase	4.700	4.459
23	MT2A	Metallothionein 2A	−1.705	−1.253
24	PGLYRP1	Peptidoglycan recognition protein 1	1.535	1.907
25	RNF182	Ring finger protein 182	3.138	3.459
26	SLC26A8	Solute carrier family 26 member 8	1.890	3.021
27	SNAI1	Snail family zinc finger 1	−2.149	−1.672
28	TECPR1	Tectonic beta-propeller repeat containing 1	−1.027	−1.240
29	THEM5	Thioesterase superfamily member 5	−1.476	−1.306
30	TNFSF11	Tumor necrosis factor superfamily member 11	−2.000	−3.000
31	YEATS2	YEATS domain containing 2	−1.631	−1.304

## Abbreviations

PS: Psoriasis
RA: Rheumatoid arthritis
PBMCs: Peripheral blood mononuclear cells
PV: Psoriasis vulgaris
PASI: Psoriasis area severe index
BSA: Body surface area
DAS28: Disease activity score in 28 joints
BMI: Body mass index
ESR: Erythrocyte sedimentation rate
CRP: C-reactive protein
RF: Rheumatoid factor
WBC: White blood cell
HGB: Hemoglobin
PLT: Platelets
IL: Interleukin
Th: T helper
Treg: Regulatory T cells
TNF: Tumor necrosis factor
CSF3: Colony-stimulating factor 3
IFN*γ*: Interferon gamma
IFNG: Interferon gene
NF-*κ*B: Nuclear factor-kappa B
MAPKs: Mitogen-activated protein kinases
ERK1/2: Extracellular signal-regulated kinase 1/2
NGS: Next-generation sequencing.
