# Supplementary material for: Cytokine Imbalance as a Common Mechanism in Both Psoriasis and Rheumatoid Arthritis
Source: Mediators Inflamm. 2017 Jan 25;2017:2405291. doi: 10.1155/2017/2405291 (PMC5296610; doi:10.1155/2017/2405291)
Supplement: Supplementary file 1 — In Supplementary Material, identified DEGs in PS vs. Control and RA vs. Control, functions and upstream regulators of the shared DEGs between PS and RA, as well as signaling pathways relevant to the merged bionetwork were listed in table in detail. [file 2405291.f1.docx]

**Supporting Tables**

**Supporting Table 1.**

**The identified differentially expressed genes in PS *vs.* Control**

| n | DEGs | Exp Fold Change |
| --- | --- | --- |
| 1 | AEBP1 | 2.615 |
| 2 | AKR1D1 | 1.132 |
| 3 | ALAS2 | -1.932 |
| 4 | ANKRD20A8P | -4.322 |
| 5 | ANXA2 | 1.237 |
| 6 | ARG1 | 1.295 |
| 7 | ATF3 | -1.179 |
| 8 | C1orf106 | 4.459 |
| 9 | C1QB | 1.153 |
| 10 | C1QC | 1.177 |
| 11 | C2CD3 | -1.334 |
| 12 | C2orf40 | 1.146 |
| 13 | C3AR1 | 1.007 |
| 14 | C4BPA | 1.504 |
| 15 | CA2 | -1.079 |
| 16 | CACNG6 | -1.329 |
| 17 | CARNS1 | -1.095 |
| 18 | CCNB2 | 1.479 |
| 19 | CD177 | 1 |
| 20 | CD302 | 4.322 |
| 21 | CELP | -1.692 |
| 22 | CEP55 | 1.303 |
| 23 | CFH | -1.355 |
| 24 | CFL2 | -1.128 |
| 25 | CHI3L1 | 1.095 |
| 26 | CISH | 1.098 |
| 27 | CLEC18B | 1.293 |
| 28 | COL5A3 | -1.246 |
| 29 | COLGALT2 | -1.237 |
| 30 | CRYBB2 | 1.14 |
| 31 | CTGF | 4.7 |
| 32 | CXXC1P1 | -1.227 |
| 33 | DAAM1 | -1.158 |
| 34 | DDX3Y | 10.07 |
| 35 | DDX43 | 1.015 |
| 36 | DSP | 1.165 |
| 37 | E4F1 | 2.174 |
| 38 | EFNA5 | -1.297 |
| 39 | EGR2 | 1.413 |
| 40 | EIF1AY | 7.937 |
| 41 | FAM177B | 1.136 |
| 42 | FHDC1 | 1.122 |
| 43 | FOLR3 | 1.787 |
| 44 | FOSB | -1.029 |
| 45 | G0S2 | -1.079 |
| 46 | GIPC3 | 1.133 |
| 47 | GSTT1 | -1.234 |
| 48 | GTF2I | -1.722 |
| 49 | HBB | -1.28 |
| 50 | HBEGF | -1.223 |
| 51 | HBM | -1.168 |
| 52 | HIST1H2BC | -2.222 |
| 53 | HLA-DQA2 | 1.548 |
| 54 | HLA-DRB4 | 1.884 |
| 55 | HLA-F | 1.316 |
| 56 | ID1 | -1.203 |
| 57 | IFI27 | -1.948 |
| 58 | IGLL3P | -5.575 |
| 59 | IL1B | -1.312 |
| 60 | ITGB4 | -1.531 |
| 61 | KDM5D | 8.299 |
| 62 | KRT1 | -1.142 |
| 63 | LGR4 | 1.103 |
| 64 | LINC00999 | -1.28 |
| 65 | LOC100505817 | -1.042 |
| 66 | LPIN3 | -1.13 |
| 67 | LTF | 1.526 |
| 68 | MCOLN3 | 1.156 |
| 69 | MET | 4.7 |
| 70 | MSH4 | 3.415 |
| 71 | MT2A | -1.705 |
| 72 | NBPF7 | -1.744 |
| 73 | NEXN | -1.058 |
| 74 | NR4A2 | -1.376 |
| 75 | NRADDP | -1.405 |
| 76 | NXF3 | -5.285 |
| 77 | PDGFRB | -1.332 |
| 78 | PGLYRP1 | 1.535 |
| 79 | PLK2 | -1.081 |
| 80 | PPIL6 | 1.1 |
| 81 | PRKY | 2.373 |
| 82 | RBP1 | -7.055 |
| 83 | REG4 | 1.722 |
| 84 | RMI2 | 1.073 |
| 85 | RNA18S5 | -1.044 |
| 86 | RNF182 | 3.138 |
| 87 | RPL27A | 1.003 |
| 88 | RPS4Y1 | 11.891 |
| 89 | SAMD14 | 1.206 |
| 90 | SERPINB2 | -1.125 |
| 91 | SFRP5 | 1.073 |
| 92 | SGCD | -1.081 |
| 93 | SLC26A8 | 1.89 |
| 94 | SLC47A1 | -1 |
| 95 | SNAI1 | -2.149 |
| 96 | SNAP47 | 2.17 |
| 97 | SORCS3 | 1.536 |
| 98 | SPNS1 | -1.034 |
| 99 | STX18-AS1 | -1.046 |
| 100 | SUPT20H | 1.059 |
| 101 | SYN1 | 4.459 |
| 102 | TCL1B | 1.304 |
| 103 | TCN1 | 1.317 |
| 104 | TECPR1 | -1.027 |
| 105 | THEM5 | -1.476 |
| 106 | TMEM45A | -1.234 |
| 107 | TMSB4Y | 1.946 |
| 108 | TNFSF11 | -2 |
| 109 | TRHDE-AS1 | 4.585 |
| 110 | TTTY14 | 1.154 |
| 111 | TXLNGY | 8.52 |
| 112 | UPK3A | -1.15 |
| 113 | USP9Y | 6.267 |
| 114 | UTY | 7.615 |
| 115 | VSTM4 | -1.678 |
| 116 | WASH1 | 1.426 |
| 117 | XK | -1.323 |
| 118 | YEATS2 | -1.631 |
| 119 | ZFY | 6.555 |
| 120 | ZSWIM4 | -1.221 |

**Supporting Table 2.**

**The identified differentially expressed genes in RA vs. Control**

| n | DEGs | Exp Fold Change |
| --- | --- | --- |
| 1 | ABTB2 | 1.209 |
| 2 | ACAA1 | 1.238 |
| 3 | ADA | 2.084 |
| 4 | ADM | 1.605 |
| 5 | AEBP1 | 2.644 |
| 6 | AHSP | 1.65 |
| 7 | ALPL | 1.852 |
| 8 | ALS2CR11 | -3.415 |
| 9 | ANKRD22 | 1.785 |
| 10 | ANO5 | 1.211 |
| 11 | ANXA3 | 1.366 |
| 12 | AREG | -1.244 |
| 13 | ARG1 | 2.224 |
| 14 | ASPM | 1.706 |
| 15 | ASPRV1 | 4.858 |
| 16 | BAMBI | 1.585 |
| 17 | BATF2 | 1.592 |
| 18 | BAX | 1.389 |
| 19 | BEX2 | 5.044 |
| 20 | BIRC5 | 1.6 |
| 21 | BMX | 4.053 |
| 22 | BPI | 2.062 |
| 23 | BTNL3 | 3 |
| 24 | BTNL8 | 1.511 |
| 25 | BUB1B | 1.285 |
| 26 | C17orf56 | 1.7 |
| 27 | C19orf71 | 1.604 |
| 28 | C1QA | 1.332 |
| 29 | C1QB | 2.356 |
| 30 | C1QC | 3 |
| 31 | C2 | 1.3 |
| 32 | C4BPA | 3.322 |
| 33 | C5orf32 | 1.28 |
| 34 | C5orf4 | 1.67 |
| 35 | C7orf25 | 2.366 |
| 36 | C8orf38 | 1.62 |
| 37 | CA1 | 1.242 |
| 38 | CA4 | 2.259 |
| 39 | CACNG6 | -1.445 |
| 40 | CCL2 | 2.072 |
| 41 | CCNA2 | 1.211 |
| 42 | CCNB1 | 1.343 |
| 43 | CCNB2 | 2.39 |
| 44 | CCR9 | 1.995 |
| 45 | CD177 | 2.404 |
| 46 | CDC45 | 1.256 |
| 47 | CDC6 | 1.423 |
| 48 | CDKN3 | 1.344 |
| 49 | CDRT15L2 | -1.704 |
| 50 | CDT1 | 1.623 |
| 51 | CEP55 | 1.554 |
| 52 | CES1 | 1.352 |
| 53 | CHI3L1 | 1.258 |
| 54 | CMPK2 | 1.294 |
| 55 | CMTM2 | 1.471 |
| 56 | CNTNAP3 | 2.273 |
| 57 | CNTNAP3B | 2.019 |
| 58 | CPNE9 | 1.277 |
| 59 | CTGF | 4.954 |
| 60 | CXCL1 | 1.819 |
| 61 | CXCR1 | 1.957 |
| 62 | CXCR2 | 1.407 |
| 63 | CXXC1P1 | -1.219 |
| 64 | CYP1A2 | -1.334 |
| 65 | CYP4F3 | 1.768 |
| 66 | DAAM1 | -1.489 |
| 67 | DDX19A | -1.263 |
| 68 | DEFA3 | 1.85 |
| 69 | DEFA4 | 1.222 |
| 70 | DHRS9 | 1.215 |
| 71 | DIRAS1 | 1.322 |
| 72 | DLGAP5 | 1.963 |
| 73 | DSCAML1 | -2.684 |
| 74 | EFCAB4B | -4.75 |
| 75 | EGR1 | 1.84 |
| 76 | EGR2 | 2.495 |
| 77 | ESPL1 | 1.229 |
| 78 | ETV7 | 1.2 |
| 79 | FAM154B | 1.55 |
| 80 | FAM177B | 1.213 |
| 81 | FCER1G | 1.477 |
| 82 | FCGR1A | 1.498 |
| 83 | FCGR3B | 3.92 |
| 84 | FFAR2 | 1.232 |
| 85 | FHDC1 | 1.631 |
| 86 | FN1 | 4.711 |
| 87 | FOLR3 | 1.853 |
| 88 | GBP1P1 | 4.954 |
| 89 | GINS2 | 1.26 |
| 90 | GSTA1 | -1.505 |
| 91 | HARBI1 | 1.539 |
| 92 | HBD | 1.396 |
| 93 | HBG1 | 1.947 |
| 94 | HBZ | 2.893 |
| 95 | HGD | -1.223 |
| 96 | HLA-DQA2 | 1.305 |
| 97 | HLA-DRB4 | 1.711 |
| 98 | HMMR | 1.247 |
| 99 | HSD17B3 | -1.238 |
| 100 | HSPA6 | 2.06 |
| 101 | IFI27 | 2.764 |
| 102 | IFI44 | 1.3 |
| 103 | IFI44L | 1.544 |
| 104 | IFIT1 | 1.81 |
| 105 | IFIT3 | 1.925 |
| 106 | IFITM3 | 1.533 |
| 107 | IGFL2 | 2.158 |
| 108 | IGLL3P | -4.353 |
| 109 | IL1R2 | 1.427 |
| 110 | INSL3 | 1.243 |
| 111 | ISG15 | 1.521 |
| 112 | ITGB4 | -2.478 |
| 113 | KCNJ15 | 1.656 |
| 114 | KCNJ2 | 1.846 |
| 115 | KCNMA1 | 2.573 |
| 116 | KIAA0101 | 1.518 |
| 117 | KIF11 | 1.273 |
| 118 | KIF4A | 1.421 |
| 119 | KIR2DL4 | 1.301 |
| 120 | KIR2DS5 | 5.09 |
| 121 | KREMEN1 | 1.504 |
| 122 | KRT1 | -1.415 |
| 123 | KRT23 | 1.71 |
| 124 | LETM2 | 1.278 |
| 125 | LIN7A | 1.296 |
| 126 | LOC100132215 | 4.392 |
| 127 | LOC100507266 | -1.27 |
| 128 | LOC642236 | 2.44 |
| 129 | LOC647012 | 4.39 |
| 130 | LRG1 | 1.418 |
| 131 | LRRC4 | 1.227 |
| 132 | LTF | 1.708 |
| 133 | MACROD2 | 1.537 |
| 134 | MCM10 | 1.626 |
| 135 | MET | 4.459 |
| 136 | METTL7B | 4.585 |
| 137 | MGAM | 1.272 |
| 138 | MIR508 | 5.64 |
| 139 | MKI67 | 1.209 |
| 140 | MME | 2.111 |
| 141 | MMP25 | 1.518 |
| 142 | MMP8 | 1.2 |
| 143 | MMP9 | 1.903 |
| 144 | MND1 | 1.585 |
| 145 | MOSC1 | 1.31 |
| 146 | MPO | 1.232 |
| 147 | MT2A | -1.253 |
| 148 | MTRNR2L1 | -1.335 |
| 149 | MX1 | 1.239 |
| 150 | MYL4 | 1.59 |
| 151 | *NCRNA00256A* | 1.22 |
| 152 | NOP16 | 2.333 |
| 153 | NOV | 2.348 |
| 154 | NRARP | -2.024 |
| 155 | OIP5 | 1.327 |
| 156 | OTOF | 2.531 |
| 157 | PBK | 1.575 |
| 158 | PGLYRP1 | 1.907 |
| 159 | PHOSPHO1 | 1.48 |
| 160 | PI4KA | 1.28 |
| 161 | PLIN4 | 2.018 |
| 162 | PLK1 | 1.303 |
| 163 | PPIL6 | 1.27 |
| 164 | PRDM16 | 2.893 |
| 165 | PRKCDBP | 1.447 |
| 166 | PRUNE2 | 1.226 |
| 167 | PSMD4 | 2.202 |
| 168 | RBP1 | -7.055 |
| 169 | RNASE1 | 1.75 |
| 170 | RNF17 | 4.807 |
| 171 | RNF182 | 3.459 |
| 172 | RPGRIP1 | 1.585 |
| 173 | RPH3A | 1.346 |
| 174 | RPL37A | 1.826 |
| 175 | RSAD2 | 1.467 |
| 176 | S100A9 | 1.341 |
| 177 | S100P | 1.468 |
| 178 | SDC3 | 1.202 |
| 179 | SELENBP1 | 1.262 |
| 180 | SERINC2 | 1.29 |
| 181 | SIGLEC1 | 2.318 |
| 182 | SIGLEC14 | 1.357 |
| 183 | SIGLEC5 | 1.627 |
| 184 | SKA1 | 1.497 |
| 185 | SLC26A8 | 3.021 |
| 186 | SLPI | 2.179 |
| 187 | SNAI1 | -1.672 |
| 188 | SNCB | 4.807 |
| 189 | SNX7 | -1.495 |
| 190 | SOCS3 | 1.357 |
| 191 | SPESP1 | 2.16 |
| 192 | STOX1 | 2.585 |
| 193 | T | 5.17 |
| 194 | TBX15 | 4.392 |
| 195 | TECPR1 | -1.24 |
| 196 | THEM5 | -1.306 |
| 197 | TIMD4 | 1.557 |
| 198 | TNFAIP6 | 1.255 |
| 199 | TNFRSF10C | 1.247 |
| 200 | TNFSF11 | -3 |
| 201 | TNNC2 | -1.258 |
| 202 | TOP2A | 1.462 |
| 203 | TPX2 | 1.9 |
| 204 | TYMS | 1.434 |
| 205 | UBB | 1.241 |
| 206 | UHRF1 | 1.924 |
| 207 | UNC13B | 1.397 |
| 208 | USP18 | 1.478 |
| 209 | WNT16 | 1.399 |
| 210 | YEATS2 | -1.304 |
| 211 | ZKSCAN4 | 4.7 |
| 212 | ZNF202 | 1.257 |

**Supporting Table 3.**

**Functions of the shared DEGs between PS and RA**

| n | Diseases or Functions Annotation | p-Value | Molecules |
| --- | --- | --- | --- |
| 1 | Cell-to-cell Signaling | 1.77E-04 | TNFSF11,CTGF,HLA-A,SNAI1,CCNB2,MET,EGR2,HLA-DRB4,LTF,C4BPA,CD177,ITGB4,CHI3L1,KRT1,EREG |
| 2 | Systemic autoimmune syndrome | 3.48E-04 | ARG1,CEACAM8,CHI3L1,HLA-A,HLA-DQA2,HLA-DRB4,LTF,MT2A,TNFSF11 |
| 3 | Cell death and apoptosis | 5.50E-04 | MT2A,TNFSF11,CTGF,HLA-A,SNAI1,RBP1,IFI27,MET,HLA-DRB4,EGR2,LTF,CD177,ITGB4,CHI3L1,EREG,ARG1 |
| 4 | Inflammatory dermatoses | 8.52E-04 | ARG1,CCNB2,CHI3L1,CTGF,EREG,HLA-A,ITGB4,LTF,MET,P2RY6,SNAI1,TNFSF11 |
| 5 | Rheumatoid arthritis | 9.23E-04 | ARG1,C1QB,CEACAM8,CHI3L1,CTGF,HLA-DQA2,HLA-DRB4,LTF,MT2A,TNFSF11 |
| 6 | Differentiation of cells | 2.08E-03 | C1QC,CHI3L1,CTGF,EGR2,EREG,KRT1,LTF,MET,RBP1,SNAI1,TNFSF11 |
| 7 | Inflammation of joint | 2.96E-03 | ARG1,CHI3L1,CTGF,HLA-DQA2,HLA-DRB4,LTF,TNFSF11 |
| 8 | Antigen presentation | 3.29E-03 | TNFSF11,HLA-DRB4,HLA-A,SNAI1 |
| 9 | Binding of cells | 6.10E-03 | CD177,HLA-A,ITGB4,KRT1,LTF,SNAI1,TNFSF11 |

**Supporting Table 4.**

**Signaling pathways relevant to the merged bionetwork**

| n | Ingenuity Canonical Pathways | -log(p-value) |
| --- | --- | --- |
| 1 | Complement System | 4.28E+00 |
| 2 | Antigen Presentation Pathway | 4.24E+00 |
| 3 | Macropinocytosis Signaling | 3.90E+00 |
| 4 | Acute Phase Response Signaling | 3.75E+00 |
| 5 | NF-κB Signaling | 2.66E+00 |
| 6 | IL-6 signaling | 2.64E+00 |
| 7 | IL-17 signaling | 2.36E+00 |
| 8 | p38 MAPK signaling | 2.05E+00 |
| 9 | chemokine signaling | 1.92E+00 |
| 10 | ILK signaling | 1.85E+00 |
| 11 | Communication between Innate and Adaptive Immune Cells | 1.74E+00 |
| 12 | phagosome maturation | 1.62E+00 |
| 13 | Cdc42 Signaling | 1.54E+00 |
| 14 | Adipogenesis pathway | 1.53E+00 |
| 15 | Epithelial Adherens Junction Signaling | 1.47E+00 |
| 16 | DNA damage-induced 14-3-3σ Signaling | 1.42E+00 |
| 17 | Crosstalk between Dendritic Cells and Natural Killer Cells | 1.34E+00 |
| 18 | Caveolar-mediated Endocytosis Signaling | 1.28E+00 |
| 19 | Dendritic Cell Maturation | 1.28E+00 |
| 20 | Regulation of the Epithelial-Mesenchymal Transition Pathway | 1.26E+00 |
| 21 | Clathrin-mediated Endocytosis Signaling | 1.22E+00 |
| 22 | Cell Cycle: G2/M DNA Damage Checkpoint Regulation | 1.02E+00 |
| 23 | Semaphorin Signaling in Neurons | 9.98E-01 |
| 24 | ATM Signaling | 9.46E-01 |
| 25 | PCP pathway | 9.25E-01 |
| 26 | Mitotic Roles of Polo-Like Kinase | 9.19E-01 |
| 27 | Role of Macrophages, Fibroblasts and Endothelial Cells in Rheumatoid Arthritis | 9.06E-01 |
| 28 | Remodeling of Epithelial Adherens Junctions | 9.00E-01 |
| 29 | VDR/RXR Activation | 8.38E-01 |
| 30 | Cyclins and Cell Cycle Regulation | 8.38E-01 |
| 31 | Renal Cell Carcinoma Signaling | 8.27E-01 |
| 32 | Altered T Cell and B Cell Signaling in Rheumatoid Arthritis | 8.17E-01 |
| 33 | FGF Signaling | 7.75E-01 |
| 34 | RANK Signaling in Osteoclasts | 7.38E-01 |
| 35 | IGF-1 Signaling | 7.11E-01 |
| 36 | Corticotropin Releasing Hormone Signaling | 7.07E-01 |
| 37 | HGF Signaling | 6.83E-01 |
| 38 | Role of Tissue Factor in Cancer | 6.66E-01 |
| 39 | OX40 Signaling Pathway | 5.18E-01 |
| 40 | Integrin Signaling | 4.53E-01 |
| 41 | Role of Osteoblasts, Osteoclasts and Chondrocytes in Rheumatoid Arthritis | 4.30E-01 |
| 42 | Axonal Guidance Signaling | 2.24E-01 |

**Supporting Table 5.**

**Upstream regulators of the shared DEGs**

| n | Upstream Regulator | Molecule Type | p-value |
| --- | --- | --- | --- |
| 1 | CSF3 | cytokine | 1.15E-06 |
| 2 | IL6 | cytokine | 2.08E-06 |
| 3 | FOS | transcription regulator | 3.24E-06 |
| 4 | P38 MAPK | group | 5.71E-06 |
| 5 | TNF | cytokine | 8.24E-05 |
| 6 | STAT3 | transcription regulator | 1.35E-04 |
| 7 | IFNG | cytokine | 1.02E-04 |
| 8 | OSM | cytokine | 0.000236 |
| 9 | IL1A | cytokine | 0.000249 |
| 10 | TGFB1 | growth factor | 0.000547 |
| 11 | IFNA2 | cytokine | 0.000627 |
| 12 | EGF | growth factor | 0.000769 |
| 13 | JUN | transcription regulator | 0.00117 |
| 14 | AKT1 | kinase | 0.00153 |
| 15 | IL1B | cytokine | 0.00215 |
| 16 | TP63 | transcription regulator | 0.00233 |
| 17 | ERBB2 | kinase | 0.00364 |
| 18 | CXCL12 | cytokine | 0.00371 |
| 19 | CD40LG | cytokine | 0.00379 |
| 20 | IL22 | cytokine | 0.00495 |
| 21 | ERK1/2 | group | 0.00541 |
| 22 | MITF | transcription regulator | 0.00612 |
| 23 | MAPK1 | kinase | 0.00828 |
| 24 | IL4 | cytokine | 0.01000 |
| 25 | EGFR | kinase | 0.0101 |
| 26 | TNFSF10 | cytokine | 0.0111 |
| 27 | IL21 | cytokine | 0.0123 |
| 28 | SMARCA4 | transcription regulator | 0.0125 |
| 29 | IL13 | cytokine | 0.0130 |
| 30 | RELA | transcription regulator | 0.0138 |
| 31 | CSF2 | cytokine | 0.0149 |
| 32 | TP53 | transcription regulator | 0.0152 |
| 33 | IL27 | cytokine | 0.0158 |
| 34 | IL15 | cytokine | 0.0174 |
| 35 | E2F1 | transcription regulator | 0.0202 |
| 36 | IL17A | cytokine | 0.0238 |
